# Novel insights into insect mediated polystyrene biodegradation through bacterial genome analyses

**DOI:** 10.1038/s41598-025-85517-x

**Published:** 2025-01-07

**Authors:** Felice Zarra, Rebecca Funari, Claudio Cucini, Francesco Nardi, Antonio Carapelli, Laura Marri, Francesco Frati

**Affiliations:** 1 Department of Life Sciences, University of Siena, 53100 Siena, Italy; 2 National Biodiversity Future Center (NBFC), 90133 Palermo, Italy

**Keywords:** Plastic degradation, Whole-genome sequencing, *Alphitobius diaperinus*, Plastivorous insect, *Stenotrophomonas indicatrix* strain DAI2m/c, Phage-like elements, Bacterial genes, Bacterial genetics, Entomology

## Abstract

Plastic pollution is a significant environmental challenge of contemporary age. Polystyrene (PS), among the most commonly used plastic polymers worldwide, is highly durable and difficult to degrade. Despite various disposal strategies, PS continues to impact biodiversity, human health, and ecosystems. Recently, the scientific community has focused on the potential role of microorganisms for plastic biodegradation, particularly those from the gut of plastivorous insects. In a previous study, three bacterial strains, each representing a distinct taxonomic group (*Klebsiella*, *Pseudomonas*, and *Stenotrophomonas*), were isolated from *Alphitobius diaperinus* larvae after rearing on a PS diet and enriched in a medium with PS as the sole carbon source. The *Stenotrophomonas* sp. strain, here identified as *S. indicatrix*, showed the greatest potential for PS degradation. The present study investigates the genetic profile of the newly isolated *S. indicatrix* strain DAI2m/c through genome sequencing, to identify enzyme-encoding genes involved in the intracellular metabolic pathways responsible for the biodegradation of the styrene monomer. Our findings indicate that the genome of *S. indicatrix* strain DAI2m/c encodes all enzymes required for one of the two recognized styrene degradation pathways, suggesting its ability to convert styrene into byproducts that are then utilized for cellular energy production.

## Introduction

Over the past decades, plastic pollution has posed a growing threat to ecosystems. Despite the well-documented toxic effects and harm inflicted on the biodiversity of our planet^1^, plastic production continues to rise, reaching a worldwide production of approximately 400.3 million tons in 2022^2^. The prevalent plastic polymers, key contributors to plastic pollution, are polyethylene (PE), polypropylene (PP), polyvinyl chloride (PVC), polyethylene terephthalate (PET), polyurethane (PU), and polystyrene (PS)^3^. Many strategies have been proposed and are used to manage the massive amount of generated Plastic Waste (PW), including landfill disposal, incineration and recycling. Not to mention that waste management is ineffective or poor across various regions globally, the majority of these strategies have been linked to detrimental environmental consequences through the release of hazardous byproducts, such as carbon dioxide, particulate matter, acidic gases, heavy metals, and the leachate derived from toxic materials that contaminate air, soil and water^4^. When PW is moved up in the environment or dumped in landfills, it slowly breaks down and undergoes gradual fragmentation into small particles known as micro-nano plastics (MNPs) which include microplastics (MPs, 1 µm-5 mm) and nanoplastics (NPs, 1-100 nm). MNPs may, in turn, accumulate in biological tissues leading to harmful consequences for human health and biodiversity alike^5,6^.

Among various plastics, polystyrene (PS) is widely used given its good mechanical properties, including its high molecular weight, strength, and durability, along with shock absorption, insulating properties, and excellent processability as well as its relative low cost. However, the qualities that make this polymer a valuable material also make it highly resistant to biodegradation. The chemical structure of PS closely resembles petroleum hydrocarbons, containing abundant alkanes and hydrophobic fragments. On these grounds, a growing body of literature has reported the capability of some microorganisms to oxidize hydrocarbons in oil-contaminated environments using alkane hydroxylases, indicating that these metabolic pathways and enzyme systems may also facilitate PS biodegradation^7–10^. Moreover, extracellular enzymes that are secreted by bacteria, including several hydrolases and monooxygenases, are known to break polymers into smaller units (oligomers, dimers, or monomers), that may be transported into the cytoplasm^11,12^.

A novel area of study has focused on symbiotic microorganisms that colonize the gut of “plastivorous insects”, that could play a role in the biodegradation processes of plastic and could in turn be deployed for remediation. Indeed, larvae of several species of Lepidoptera and Coleoptera, such as *Galleria mellonella, Plesiophthalmus davidis, Tenebrio molitor, Tenebrio obscurus, Tribolium castaneum* and *Zophobas atratus*, have shown encouraging abilities for PS biodegradation^7^. This degradation process is influenced by several factors, including the mechanical breakdown of plastic through mastication and ingestion, which increases the plastic surface area available for microbial colonization, and, additionally, the specific conditions within the gut environment that facilitate microbial activity. Although, the substantial contribution is provided by symbiotic bacteria present in their gut^7^. Among these, multiple strains have already shown PS biodegradation capabilities, such as *Exiguobacterium* sp. (strain YT2), a symbiont of the *T. molitor*’s midgut^13^, and *Acinetobacter* sp. AnTc-1 isolated from the gut of *T. castaneum*^14^. Additionally, a recent study demonstrated that *Pseudomonas* sp. DSM 50071, isolated from the gut of *Z. atratus*, efficiently biodegraded PS through the secretion of extracellular hydrolase enzymes^15^.

**Fig. 1:**
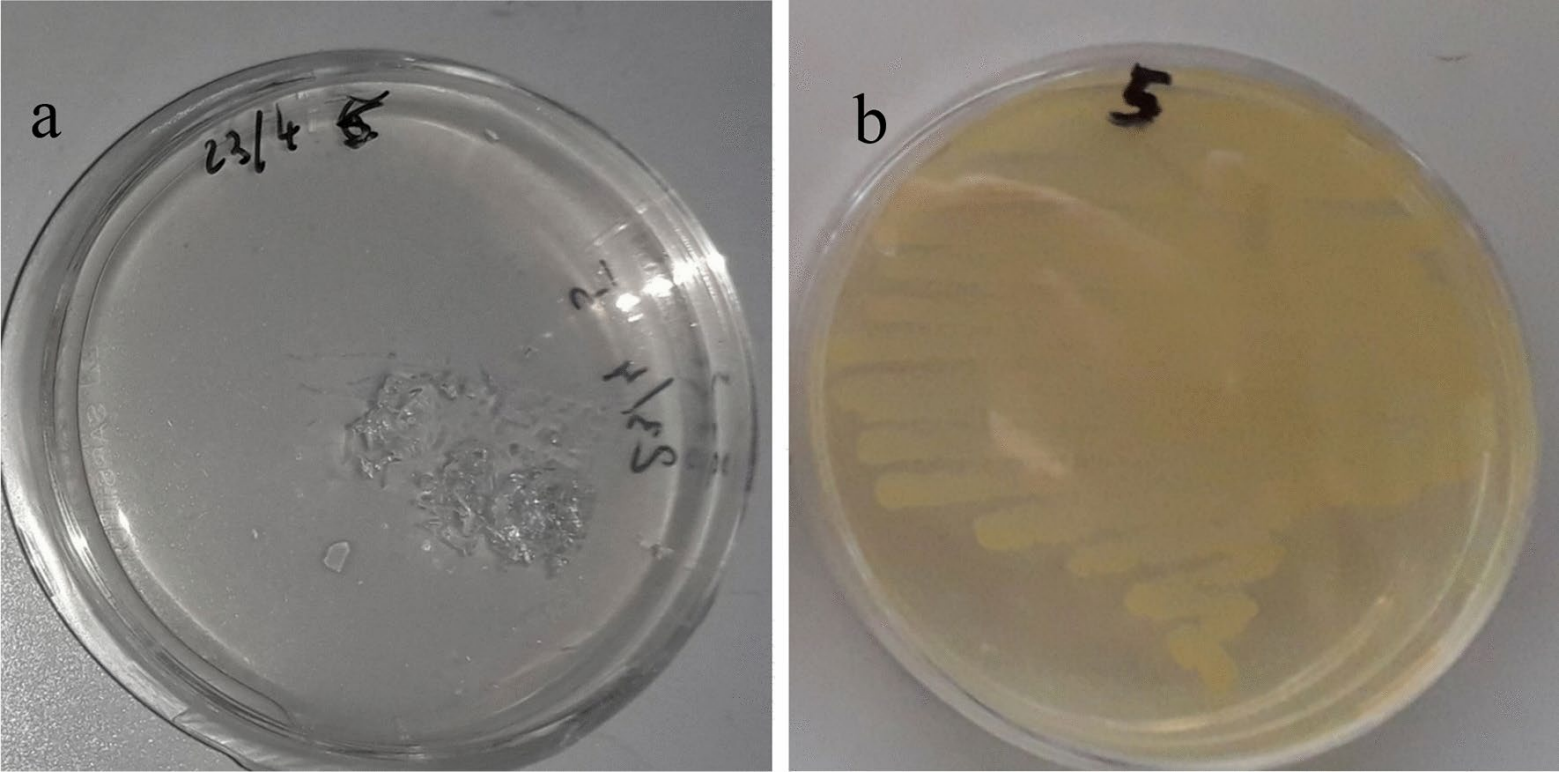
PS film degradation and bacterial growth on agar plates. (**a**) PS film appearance on liquid carbon-free basal medium (LCFB) agar plates after 1 year storage at 4 °C. A PS film (10 mm × 10 mm; ~ 9–10 mg) was transferred from 28 days-old *Stenotrophomona*s sp. culture in LCFB at the conclusion of the experiments reported by Cucini et al.^17^. (**b**) Growth on LB agar plate after 48 h at 27 °C of the sample collected by rubbing PS film remains with a sterile cotton swab.

Another insect with promising degradative abilities is *Alphitobius diaperinus*, a member of the Tenebrionidae family. Previous research has reported that feeding with PS foam resulted in substantial changes to the gut bacterial microbiome^16^. Subsequently, the gut bacterial population associated with the plastic-eating *A. diaperinus* was analysed after an enrichment phase, in a medium with limited chemical composition and PS as the sole carbon source, in the first reported study integrating culture-dependent and molecular analyses^17^. The results indicated the predominance of *Klebsiella*, *Pseudomonas*, and *Stenotrophomonas* in both cases. When the bacterial isolates of each taxon were grown as monoculture in a synthetic medium with PS, an increase in the number of viable cells found attached to PS films was observed for all three bacteria, but statistical significance was reached only by the *Stenotrophomonas* sp. Isolate^17^. Moreover, over a prolonged incubation period, this strain produced a visible PS deterioration (Fig. 1.a). With these premises, a thorough study was undertaken through genome sequencing, specifically examining the species’ genetic arsenal for styrene degradation and the putative metabolic pathways involved in the process. Additionally, a comprehensive phylogenetic analysis was performed to accurately classify the bacterial strain and explore its evolutionary relationships with related lineages. In turn, our analyses provide valuable insights into the genetic basis of *S. indicatrix* capability to degrade PS, improving our understanding of microbial degradation mechanisms in environmental microbiology with an outlook at possible biotechnological applications.

## Results

### Genome features

The almost complete sequence of the *Stenotrophomonas* sp. 2 m/c 16S rDNA gene (1,497 bp), obtained with Sanger sequencing and further confirmed through genome sequencing, showed 100% identity with *Stenotrophomonas indicatrix* strain DAIF1 (CP037883.1)^18^; based on this observation, the strain under study is hereafter referred to as *S. indicatrix* strain DAI2m/c.

**Fig. 2:**
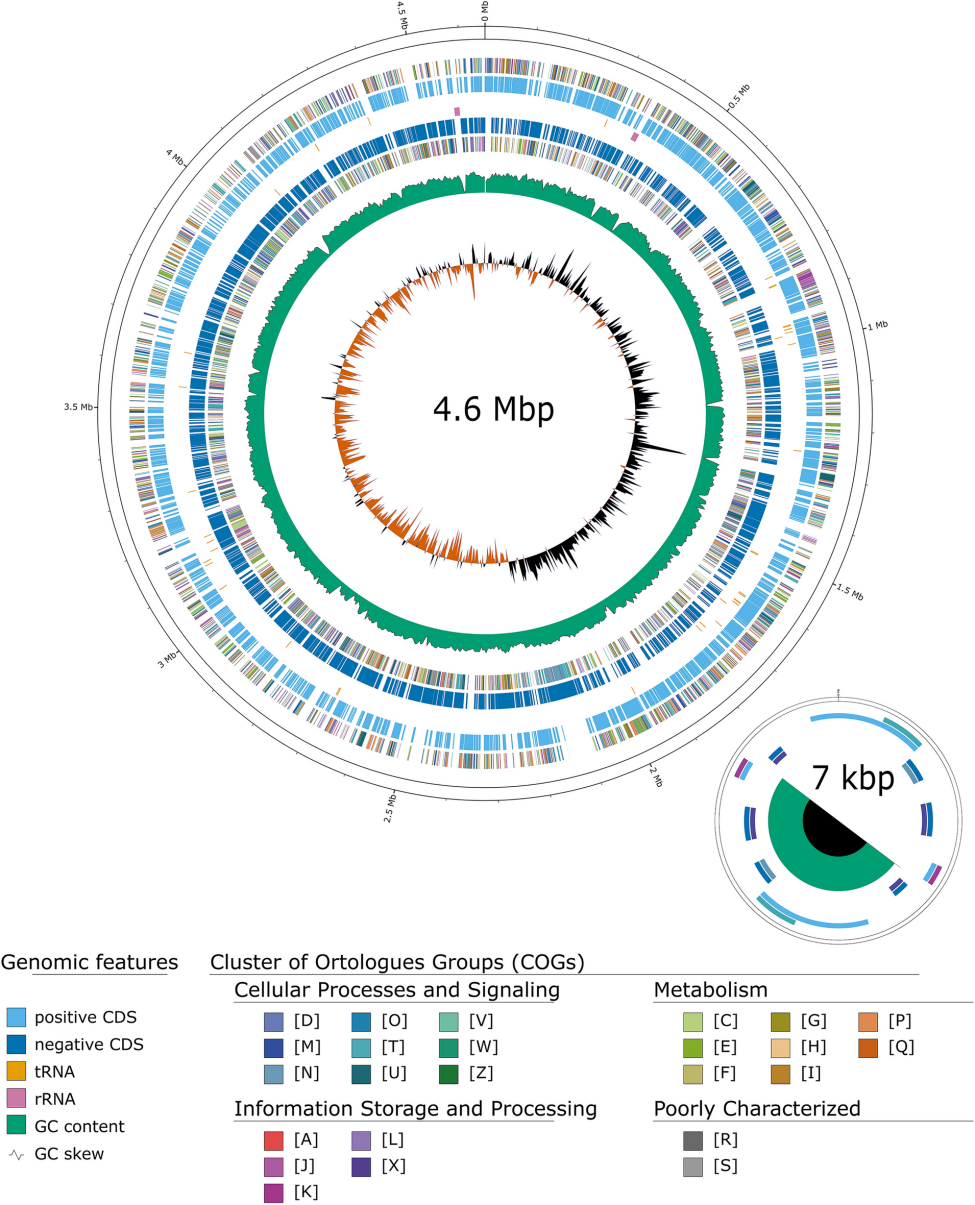
Circular representation of the chromosome and plasmid of *Stenotrophomonas indicatrix* strain DAI2m/c, including gene and functional regions (outer circles). GC content and skew values are displayed in the two innermost circles.

Illumina DNA sequencing produced a total of 9.57 million read pairs, while ONT long-read sequencing generated 354.5 thousand reads, both corresponding to a coverage of 100% reads coverage. The assembly resulted in a single circular molecule of 4,651,465 bp with a GC content of 65.76%, along with a plasmid of 7,081 bp exhibiting a GC content of 43.39% (Fig. 2, Table 1). The BUSCO statistics indicated a high level of completeness (99.7%), with a small fraction of duplication and missingness (0.2% and 0.1%, respectively), while no fragmentation was reported. The PHASTEST analysis identified phage-related genes sequences in approximately 98% of the total length of the extrachromosomal DNA (Supplementary Fig. 1). At the annotation level, identity was found between chromosome sequences of DAI2m/c and holin S, (pgaptmp_000287) and endolysin R (pgaptmp_000368, pgaptmp_000540, pgaptmp_001024, pgaptmp_001183, pgaptmp_001870, pgaptmp_002081, pgaptmp 002083, pgaptmp_002084, pgaptmp 002088, pgaptmp_002916, pgaptmp_003288). Moreover, sequences sharing identity with endolysin R (pgaptmp_004233, pgaptmp_004240), and endopeptidase Rz (pgaptmp_004241, pgaptmp_004234) were found on the plasmid together with two partially complete sequences sharing identity with holin S (pgaptmp_004232, pgaptmp_004239).

**Table 1:** Genomic features of *Stenotrophomonas indicatrix* strain DAI2m/c*.*

Features	Chromosome	Plasmide-like DNA
Number of circular contigs	2	
Coverage	100%	
N50/L50 (Mb)	4.651/4.651	
‍Single complete BUSCO	99.7%	
‍Single duplicated BUSCO	0.2%	
‍Fragmented BUSCO	0%	
Missing BUSCO‍	0.1%	
Total size (bp)	4,651,465	7,081
GC content	65.75%	43.39%
Genes (total)	4,245	14
CDSs (total)	4,158	14
Genes (coding)	4,123	14
CDSs (with protein)	4,123	14
Genes (RNA)	87	–
rRNAs	5, 4, 4 (5S, 16S, 23S)	–
Complete rRNAs	5, 4, 4 (5S, 16S, 23S)	–
tRNAs	70	–
ncRNAs	4	–
Pseudo genes (total)	35	2
CDSs (without protein)	35	2
Pseudo genes (ambiguous residues)	0 of 35	0 of 2
Pseudo genes (frameshifted)	17 of 35	0 of 2
Pseudo genes (incomplete)	20 of 35	0 of 2
Pseudo genes (internal stop)	6 of 35	2
Pseudo genes (multiple problems)	6 of 35	–

### Styrene degradation pathway

The search for orthologs based on the eggNOG annotation of the *S. indicatrix* DAI2m/c genome revealed the presence of five genes encoding for enzymes involved in the styrene degradation pathway (Supplementary Fig. 2a): phenylacetaldehyde dehydrogenase [EC:1.2.1.39], homogentisate 1,2-dioxygenase [EC:1.13.11.5], maleylacetoacetate isomerase [EC:5.2.1.2], fumarylacetoacetase [EC:3.7.1.2], 2-oxopent-4-enoate hydratase [EC:4.2.1.80]. Additionally, manual curation based on Pfam domains identified genes for six additional enzymes in the *S. indicatrix* DAI2m/c genome which exhibit a domain composition characteristic of enzymes related to styrene degradation (Supplementary Fig. 2b): cis-1,2-dihydrobenzene-1,2-diol dehydrogenase [EC:1.3.1.19], catechol 2,3-dioxygenase [EC:1.13.11.2], 2-hydroxymuconate-semialdehyde hydrolase [EC:3.7.1.9], glutaconate CoA-transferase, subunit A [EC:2.8.3.12], lactoyl-CoA dehydratase subunit alpha [EC:4.2.1.54], propionate CoA-transferase [EC:2.8.3.1].

However, during the manual annotation process, few enzymes were characterized by multiple Pfam profiles: two oxidoreductases with incomplete classification [EC:1.14.12.-; EC:1.14.13.-], and 4-hydroxy-2-oxovalerate aldolase [EC:4.1.3.39]. The search of these domains within the *S. indicatrix* DAI2m/c genome, retrieved only a partial ortholog correspondence (five domains out of 10, 21 domains out of 40, and two domains out of three, respectively). Although it was not entirely possible to associate these genetic features to *S. indicatrix* DAI2m/c orthologs, a good number of Pfam domains were identified, suggesting that these functions might indeed be encoded by genes within the *S. indicatrix* DAI2m/c genome. All Pfam profiles used for manual annotation are available in Supplementary Table S1. Styrene monooxygenase [EC:1.14.14.11], styrene-oxide isomerase [EC:5.3.99.7] and phenylacetate 2-hydroxylase [EC:1.14.14.54] were not found, neither on the eggNOG annotation, nor following manual inspection of Pfam domains.

**Fig. 3:**
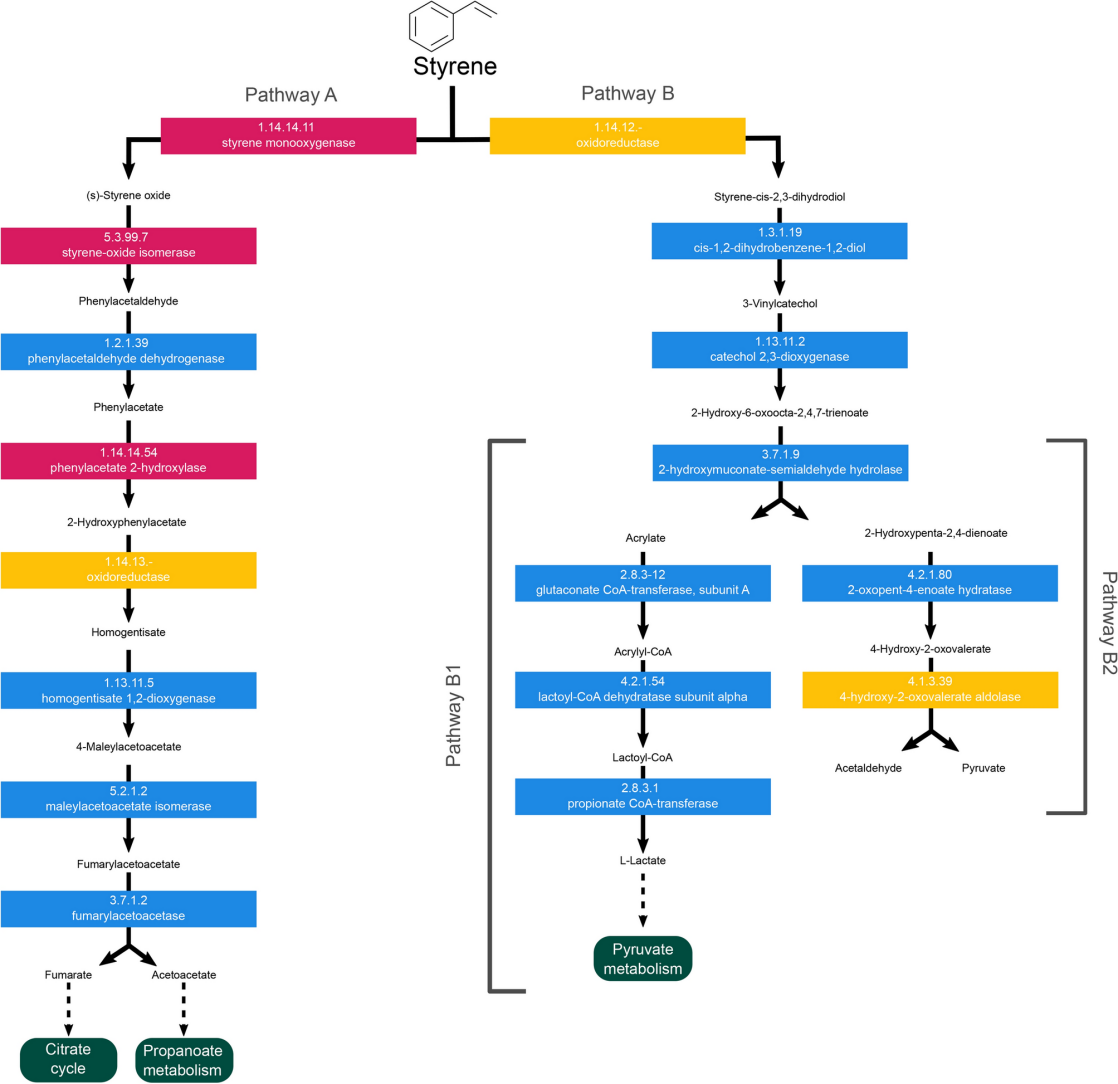
Representation of the styrene degradation pathway in *Stenotrophomonas indicatrix* strain DAI2m/c following the original map00634 available in the Kyoto Encyclopedia of Genes and Genomes (KEGG) pathway database, and the styrene pathway map presents in the EAWAG biocatalysis/biodegradation database (BBD). This styrene map reports the integration of automatic and manual annotations. The presence and absence of styrene map-related enzymes are represented by blue and pink boxes, respectively. The enzymes with partial retrieval of Pfam profiles are indicated by yellow boxes.

All results are summarized in a single figure that integrates both automatic and manual annotations, displaying information on the presence/absence and the specific names of enzymes (Fig. 3).

The comparative genome analysis conducted with BRIG, using *S. indicatrix* strain DAIF1 as a reference, revealed the presence of the same enzyme-encoding genes involved in the styrene degradation pathway in both strains. *Stenotrophomonas indicatrix* DAI2m/c genes exhibited a percentage identity ranging from 91 to 98% when compared to those of *S. indicatrix* DAIF1 (Supplementary Fig. 3).

### Phylogenetic analysis

**Fig. 4:**
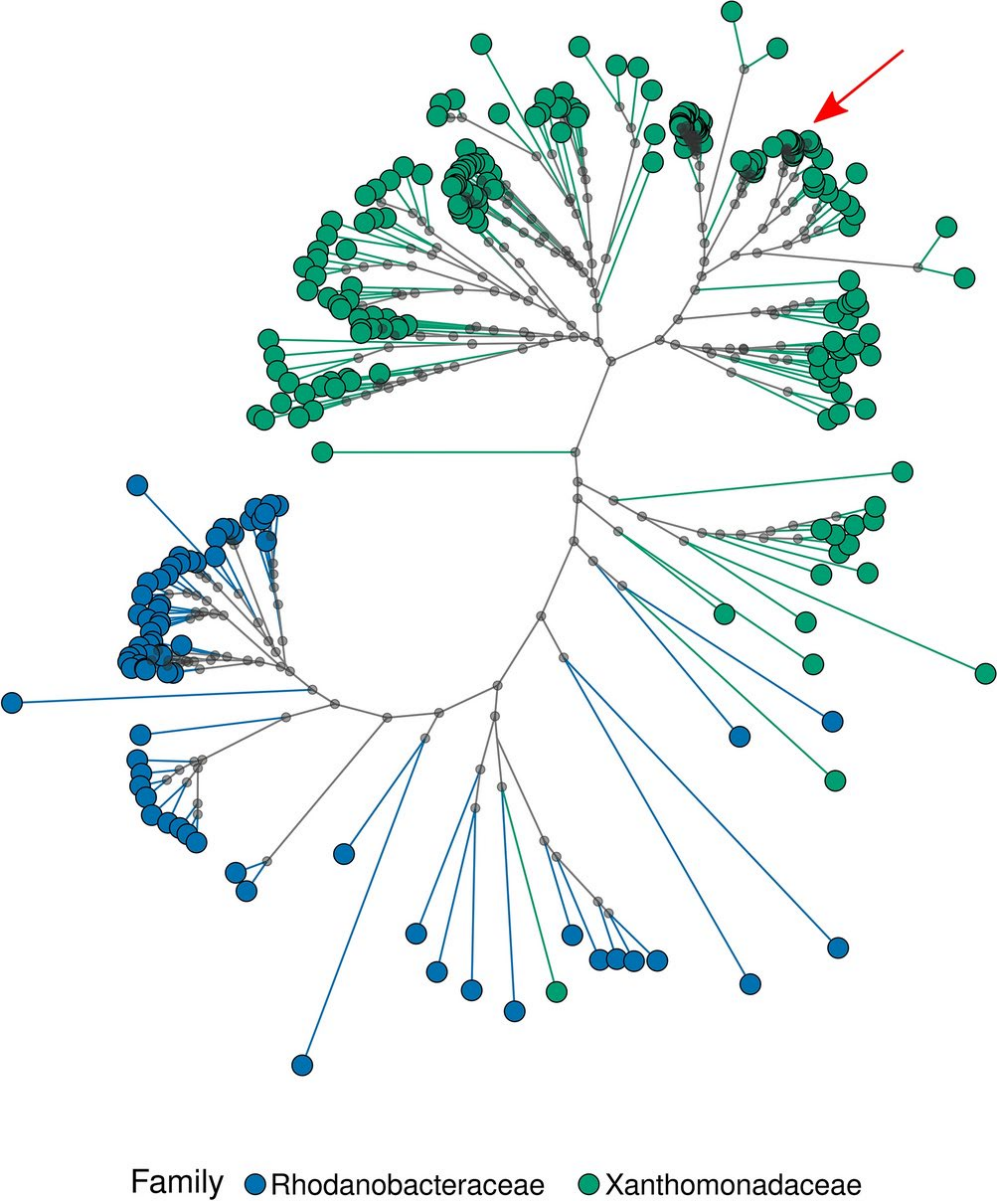
Unrooted phylogenetic reconstruction of the order Xanthomonadales using BUSCO scOGs. The two families are colour coded. Supported nodes (UFB > 95) are identified with a grey dot. Colours reflect family taxonomic position. The red arrow indicates the position of *S. indicatrix* strain DAI2m/c position. A more detailed representation is provided in the Supplementary Fig. 4.

The phylogenetic analysis of all the available Xanthomonadales species, based on a supermatrix of 368,075 amino acid positions, supported the monophyly of Xanthomonadaceae within a paraphyletic Rhodanobacteraceae (Fig. 4, see Supplementary Fig. 4 for a more detailed visualization). In fact, the bulk of Xantomonadaceae is recovered as sister group to one specific group of Rhodanobacteraceae, namely *Chyayiivirga flava* and *Aquimonas varaii*. Incidentally, two species belonging to the Xanthomonadaceae, *Pseudolysobacter antarcticus* and *Pseudomarinomonas arenosa*, were further identified as outliers (Supplementary Fig. 4). In turn, a relatively conserved and well supported cluster was identified that corresponds to the *Stenotrophomonas* genus, with 26 out of 27 species belonging to the genus. The only exception was *S. panachiumi* which was recovered within the *Xanthomonas* phylogroup, raising doubts on the actual taxonomic position of this species. The newly sequenced *S. indicatrix* strain DAI2m/c was correctly retrieved within family Xanthomonadaceae and within genus *Stenotrophomonas*, closely related to *S. indicatrix* DAIF1 (GCF_004551575.1), and also associated with *S. lactitubi* (GCF_900188015.1) within the genus *Stenotrophomonas*.

## Discussion

In natural environments PS biodegradation is notably inefficient due to a multitude of biotic and abiotic factors, as well as the intrinsic properties of the polymer and the additives incorporated into the plastic^7^; therefore, it often takes decades or even centuries to complete. The persistent nature of PS results in significant pollution, health issues, and ecological disturbances^8^. Microorganisms have been demonstrated to play a crucial role in the biodegradation of PS, and the ability of their enzymes to degrade petroleum hydrocarbon alkanes of various chain lengths has been extensively documented in the literature^7–10^. Given the structural similarities between petroleum hydrocarbons and the PS polymer, bacterial enzymes known to be involved in alkane metabolic pathways, such as alkane 1-monooxygenase, cytochrome P450, or alkane hydroxylase, can also participate in the extracellular depolymerization of PS^12^.

Recent studies have focused on extracellular mechanisms responsible for the degradation of the PS polymers into oligo- and monomers^11,12^ but did not account for the secondary degradation processes that occur once these molecules are uptaken by the cell. In order to fill this gap, the present study delves into the intracellular mechanisms that occur within the newly isolated strain *S. indicatrix* DAI2m/c to shed light on how styrene monomers are incorporated into metabolic pathways.

The genus *Stenotrophomonas* is a member of the family Xanthomonadaceae within the order Xanthomonadales, an early diverging group of Gammaproteobacteria^19^. Xanthomonadales are divided into two families: Xanthomonadaceae (or Lysobacteriaceae) and Rhodanobacteraceae^20^. The phylogenetic analysis using BUSCO scOGs supported a paraphyletic condition for the Rhodanobacteriaceae whereas Xanthomonadaceae were identified as monophyletic, excluding the enigmatic positions of two poorly studied taxa: *P. antarcticus* and *P. arenosa*. The first species, formally belonging to the Xanthomonadaceae family^21^, was recently proposed by some authors^20^ as to be re-assigned to the Rhodanobacteriaceae. To the best of our knowledge, no systematic and/or phylogenetic data are available, in turn, for *P. arenosa*. The phylogenetic analysis also allowed to clarify the systematic position of the newly identified *S. indicatrix* DAI2m/c as part of the *Stenotrophomonas* genus (Fig. 4, Supplementary Fig. 4).

As described in the KEGG pathway database (entry: map00643) and the BBD Styrene Pathway Map, styrene monomers, following uptake, enter two primary metabolic pathways, identified in this study as pathway A and pathway B (Fig. 3). In pathway A, styrene monooxygenase [EC:1.14.14.11] is the first enzyme that interacts with the styrene monomer. The activity of a cascade of seven additional enzymes results in the formation of two final by-products of styrene metabolism, fumarate and acetoacetate, which enter as substrates in the citric acid cycle and the propanoate metabolism, respectively. In pathway B, an oxidoreductase [EC 1.14.12.-] converts the styrene monomer into styrene-cis-2,3-dihydrodiol. This intermediate is further processed by three enzymes to produce acrylate and 2-hydroxypenta-2,4-dienoate. In sub-pathway B1, acrylate is converted into L-lactate by the action of three enzymes, which then enters pyruvate metabolism. In sub-pathway B2, 2-hydroxypenta-2,4-dienoate is converted by the activity of two enzymes into acetaldehyde and pyruvate as end by-products. The latter are directly implicated in multiple subsequent metabolic pathways.

The automatic functional annotation revealed the presence of five enzyme-encoding genes in the styrene biodegradation pathway, four of which pertaining to pathway A and one to pathway B (Fig. 3, Supplementary Fig. 2a). Subsequent manual curation of annotations using Pfam profiles confirmed the attribution of those enzymes and led to the identification of six supplementary enzymes pertaining to pathway B (Fig. 3, Supplementary Fig. 2b). Finally, the 4-hydroxy-2-oxovalerate aldolase [EC:4.1.3.39] and two oxidoreductases [EC:1.14.12.-; 1.14.13.-] were partially identified due to their protein domain unspecificity. Indeed, only a proportion of characteristic Pfam domains were retrieved in *S. indicatrix* DAI2m/c genome (66.7%, 50.0%, and 52.5%, respectively).

Genes encoding for all enzymes in styrene biodegradation pathway B were identified in the chromosome of *S. indicatrix* DAI2m/c, while only five out of eight were identified in pathway A (Fig. 3). As such, pathway B, including sub-pathways B1 and B2, appears to be fully viable as the primary metabolic pathway involved in styrene degradation in *S. indicatrix* DAI2m/c, capable of transforming the styrene monomer into by-products that subsequently enter canonical energy-production biochemical pathways. Given the presence of five out of eight genes, it cannot nevertheless be excluded that pathway A is also viable in *S. indicatrix* DAI2m/c, with alternative enzymes that may recover the functionality of the three non-identified enzymes, but this hypothesis needs further confirmation.

The comparative genome analysis conducted between *S. indicatrix* DAI2m/c and *S. indicatrix* DAIF1 facilitated the identification of genomic similarities. Enzyme-encoding genes of styrene map present in both strains exhibit a small identity variation between the two strains (Supplementary Fig. 3). Furthermore, *S. indicatrix* DAI2m/c, unlike *S. indicatrix* DAIF1, harbours a plasmid carrying many phage-like elements (Supplementary Fig. 1). Phages and their remnants have been identified for a long time as significant contributors to the development of biofilms across several genera^22,23^. Significantly, the lysis genes of defective Lambda prophage DLP12 have been shown to be important for curli fiber production, which, due to their adhesive properties and involvement in cell–cell and cell-surface interactions, are pivotal for the biofilm formation in *Escherichia coli* K-12^23^. DLP12 lysis genes show high homology to their counterparts that are present in the SRRz lysis gene cassette from bacteriophage Lambda^24^. The presence of similar viral lysis sequences in several locations of *S. indicatrix* DAI2m/c genome might then contribute to the release of the DNA necessary for a proper biofilm development on a polystyrene surface. It has indeed been demonstrated that extracellular DNA is a common feature of biofilms formed by many bacterial species; it was reported that the activity of a cryptic prophage endolysin was essential for the biogenesis of bacterial membrane vesicles and biofilms in *Pseudomonas aeruginosa*^25^, and in the last decade, articles have followed one another reporting prophages that enhance biofilm formation by facilitating DNA release^26^.

The main outcome of this study is the identification of *S. indicatrix* strain DAI2m/c as a potential candidate for styrene degradation. The genomic and bioinformatic analyses not only described its genome, but also revealed previously unreported intracellular pathways linked to styrene degradation in this bacterium. The findings of this study establish a strong foundation for future research aimed at generating quantitative data on styrene degradation and conducting genetic experiments to expand our understanding on the functional roles of these identified styrene degradation genes. Future research could also explore extracellular enzymes that can target the entire polystyrene chain and conduct further metabarcoding analyses of the gut microbiota in polystyrene-feeding insects to study its composition under various experimental conditions. Such experiments could provide deeper insights into the full biodegradation potential of *S. indicatrix* strain DAI2m/c, enhance the understanding of microbial degradation processes, and help to identify other potential candidates for polystyrene bioremediation.

## Methods

### Growth conditions, microorganism, and molecular fingerprinting

Luria Bertani broth (LB) (1% Bacto tryptone, 0.5% yeast extract, 0.5% NaCl, distilled water, pH 7.2), and LB agar (LB added with 1.5% agar) were used for routine culture maintenance unless otherwise stated.

The bacterial strain that is the focus of this study was isolated after an enrichment phase in a medium with limited chemical composition and PS as sole carbon source from PS-fed larvae of *A. diaperinus*^17^, and it was initially reported as *Stenotrophomona*s sp. 2 m/c based on the partial sequence of the 16S rDNA gene (474 bp)^17^. Upon observing as a serendipitous finding the partial deterioration of a PS film with attached 2 m/c cells (~ 9.0 × 10^5^) after one year storage (Fig. 1a), a sterile cotton swab was carefully rubbed over the entire surface of the PS film remains and used to streak a LB agar plate. The resulting yellow pigmented growth (Fig. 1b) likely indicated a pure culture of *Stenotrophomona*s sp. DAI2m/c. Yellow pigmentation was one of the characteristics previously reported for the colony morphotype shown by DAI2m/c on LB agar; moreover, by repeating the primary phenotypic tests previously described^17^, the strain was confirmed as aerobic/ facultative anaerobic, non-glucose fermenting, non-lactose fermenting and oxidase negative^17^.

To obtain the full-lenght 16S rDNA gene sequence, an individual colony of *Stenotrophomona*s sp. strain DAI2m/c was picked from the LB agar plate, suspended in 50 µl of sterile double-distilled water, and incubated for 5 min at 100 °C. The 16S rDNA gene was amplified using eubacterial universal primers and sequenced with Sanger method on both strands at BMR Genomics (Padua, Italy)^27^. Sequences were analysed using the BLAST algorithm^28^. The updated nucleotide sequence data (1,497 bp) was submitted to the DDB/EMBL/GenBank database under the accession number OL470964.2. The strain has also been deposited in the “Centre de Ressources Biologiques de l’Institut Pasteur” CRBIP and in the “Leibniz Institute DSMZ” under accession number CIP 112583 and DSM 120327, respectively.

### Genomic DNA extraction and sequencing

Genomic DNA of the *Stenotrophomonas* sp. 2 m/c was extracted using the Wizard® SV Genomic DNA Purification System (Promega Corporation, Madison, WI, USA) according to manufacturer’s instructions. Final DNA concentration was determined using Qubit fluorometer 4.0 (Invitrogen Ltd., Thermo Fisher Scientific, Singapore) with Qubit 1X dsDNA HS Assay Kit (Invitrogen Ltd., Thermo Fisher Scientific, Eugene, Oregon, USA). DNA quality was evaluated through Thermo Scientific™ NanoDrop™ One/OneC Microvolume UV–Vis Spectrophotometer (NanoDrop Technologies, Madison, WI, USA) and 1% agarose gel electrophoresis. The DNA extraction was used for Illumina short-read and Oxford Nanopore Technology (ONT) long-read sequencing at IGATech (Udine, Italy). For short-read sequencing, DNA libraries were prepared using Celero™ DNA-Seq kit (NuGEN, San Carlos, CA) following the manufacturer’s instructions. After quantification by Qubit 2.0 Fluorometer (Invitrogen, Carlsbad, CA) and quality test by Agilent 2100 Bioanalyzer High Sensitivity DNA assay (Agilent technologies, Santa Clara, CA), DNA libraries were sequenced on a NovaSeq6000 (Illumina, Inc) in paired-end 150 bp mode. For long-read sequencing, DNA libraries were prepared using the SQK-LSK114 ligation kit and sequenced with a PromethION (Oxford Nanopore) machine on a FLO-PRO114M flow cell.

### Genome assembly and annotation

Illumina raw reads were processed for base calling, demultiplexing and adapter masking with Illumina BCL Convert v3.9.3. Trimming was performed using fastp v.0.23.2^29^ under default settings. ONT reads were filtered using Filtlong v.0.2.1 (*keep_percent* parameter: 90%; available at: https://github.com/rrwick/Filtlong). Filtered ONT reads were employed for a de novo genome assembly using Flye v.2.8.1^30^ with five iterations and plasmid assembly rescue option. The resulting assembly was then oriented to the *dnaA* gene using Circlator v.1.5.5^31^. To enhance accuracy and quality, this first assembly underwent genome polishing via three iterations of Pilon v.1.24^32^ using BWA as mapper^33^*.* General assembly statistics were tabulated using stats.sh (available in the BBtools suite^34^), while a graphical representation of the genome was achieved using GenoVi^35^. BUSCO v.5.4.4 was used to assess the completeness of the final genome assembly against the closest available database, i.e. *xanthomonadales_odb10*^36^. The whole genome was annotated using the NCBI Prokaryotic Genome Annotation Pipeline (PGAP) v.2024–07-18^37^ and eggNOG v.5.0^38^. The web server PHASTEST (PHAge Search Tool with Enhanced Sequence Translation) was used for the identification of prophage sequences within the bacterial genome^39^. The origin of replication of the extrachromosomal element (oriV) was identified using Ori-Finder^40^. BLAST algorithm^28^ was applied in order to align genome annotations with a bacteriophage Lambda synthetic construct (KY609225) comprising the SRRz lysis gene cassette from bacteriophage Lambda that encodes holin (S, essD or ybcR), endolysin (R or ybcS), and endopeptidase (Rz or ybcT)^24^.

### Styrene degradation pathway

The list of orthologous enzymes involved in the canonical styrene degradation pathway (entry: map00643) was retrieved from the Kyoto Encyclopedia of Genes and Genomes (KEGG) pathway database. Initially, genes with a corresponding EC number in the *Stenotrophomona*s sp. 2 m/c eggNOG functional annotation were extracted. Subsequently, each enzyme from the KEGG styrene degradation pathway was individually taken into account in a manual curation to: (a) confirm in silico attributions; and (b) identify additional potential orthologs among non-annotated enzymes based on the presence of the characteristic Pfam domains. Briefly, EC numbers related to the styrene degradation pathway were explored in the UniProtKB/Swiss-Prot database, filtering results for the kingdom Bacteria, and the associated Pfam domains were retrieved (Supplementary Table 1). Each Pfam profile was independently pressed (hmmpress) and scanned (hmmscan) against the translated coding sequences (CDS) of *S. indicatrix* using HMMER v.3.3.2^41^ with an E-value threshold of 1e−05.

To gather further details on the processes involved in the biological breakdown of styrene, and to complement the KEGG pathway map, the same procedures were performed starting from the EAWAG Biocatalysis/Biodegradation Database (BBD) Styrene Pathway Map^42^, studied and described in *Rhodococcus rhodochrous* NCIMB 13259^43^. This integration provided a more detailed representation of the styrene degradation pathway.

In parallel, a comparative genome analysis between *Stenotrophomona*s sp. 2 m/c and the reference genome of *Stenotrophomonas indicatrix* (strain DAIF1, NCBI accession number: CP037883.1) ^18^, was carried out using BLAST Ring Image Generator (BRIG) v. 0.95^44^. This analysis allowed for the visualization and comparison of genomic identity between the two strains, with a particular focus on enzyme-encoding genes of interest.

### Phylogenetic analysis

All RefSeq reference genomes of Xanthomonadales (also known as Lysobacteriales) available in the NCBI-Genome database as of March 2024 (n = 274) were downloaded and processed in a BUSCO completeness analysis v.5.4.4 using the lineage *xanthomonadales_odb10* as reference*.* Single-copy orthologs (scOGs; n = 1157) from all genomes were extracted and individually aligned with mafft v. 7.475 using the *–auto* parameter^45^. Alignments were trimmed via trimAl^46^ (--*automated1*) and combined in a single super-matrix with catsequences (available at: https://github.com/ChrisCreevey/catsequences). A phylogenetic tree was built using IQTREE2 v.2.2.0^47^ (*-B 2000 -m MFP*) and visualized/edited using the ggtree library v3.12.0 in the R environment^48^.

## Supplementary Information

Supplementary Figures.

Supplementary Tables.

## Data Availability

All supporting data, code and protocols have been provided within the article, through supplementary data files or on the following link: https://github.com/ESZlab/Stenotrophomonas_indicatrix_WGS. This repository contains codes used for genome assembly and annotation. Raw read data was uploaded to SRA: SRR29926346, SRR29926347 under the BioProject PRJNA1139115. *Stenotrophomonas indicatrix* genome was submitted to GenBank using the following accession number: CP168152.1, CP168153.1. The strain has been deposited in the "Centre de Ressources Biologiques de l’Institut Pasteur" (CRBIP) under accession number CIP 112583, and in the "Leibniz Institute DSMZ" under accession number DSM 120327.
