# Correction: Novel insights into insect mediated polystyrene biodegradation through bacterial genome analyses

**DOI:** 10.1038/s41598-025-22322-6

**Published:** 2025-10-08

**Authors:** Felice Zarra, Rebecca Funari, Claudio Cucini, Francesco Nardi, Antonio Carapelli, Laura Marri, Francesco Frati

**Affiliations:** 1 Department of Life Sciences, University of Siena, 53100 Siena, Italy; 2 National Biodiversity Future Center (NBFC), 90133 Palermo, Italy

Correction to: *Scientific Reports* 10.1038/s41598-025-85517-x, published online 07 January 2025

The original version of this Article contained errors.

In the Methods section, under the subheading ‘Growth conditions, microorganism, and molecular fingerprinting’,

“To obtain the full-lenght 16S rDNA gene sequence, an individual colony of *Stenotrophomona*s sp. strain DAI2m/c was picked from the LB agar plate, suspended in 50 µl of sterile double-distilled water, and incubated for 5 min at 100 °C. The 16S rDNA gene was amplified using eubacterial universal primers and sequenced with Sanger method on both strands at BMR Genomics (Padua, Italy)^27^. Sequences were analysed using the BLAST algorithm^28^. The updated nucleotide sequence data (1,497 bp) was submitted to the DDB/EMBL/GenBank database under the accession number OL470964.2.”

now reads:

“To obtain the full-lenght 16S rDNA gene sequence, an individual colony of *Stenotrophomona*s sp. strain DAI2m/c was picked from the LB agar plate, suspended in 50 µl of sterile double-distilled water, and incubated for 5 min at 100 °C. The 16S rDNA gene was amplified using eubacterial universal primers and sequenced with Sanger method on both strands at BMR Genomics (Padua, Italy)^27^. Sequences were analysed using the BLAST algorithm^28^. The updated nucleotide sequence data (1,497 bp) was submitted to the DDB/EMBL/GenBank database under the accession number OL470964.2. The strain has also been deposited in the “Centre de Ressources Biologiques de l’Institut Pasteur” CRBIP and in the “Leibniz Institute DSMZ” under accession number CIP 112583 and DSM 120327, respectively.”

In addition, in the Data Availability section,

“All supporting data, code and protocols have been provided within the article, through supplementary data files or on the following link: https://github.com/ESZlab/Stenotrophomonas_indicatrix_WGS. This repository contains codes used for genome assembly and annotation. Raw read data was uploaded to SRA: SRR29926346, SRR29926347 under the BioProject PRJNA1139115. *Stenotrophomonas indicatrix* genome was submitted to GenBank using the following accession number: CP168152.1, CP168153.1.”

now reads:

“All supporting data, code and protocols have been provided within the article, through supplementary data files or on the following link: https://github.com/ESZlab/Stenotrophomonas_indicatrix_WGS. This repository contains codes used for genome assembly and annotation. Raw read data was uploaded to SRA: SRR29926346, SRR29926347 under the BioProject PRJNA1139115. *Stenotrophomonas indicatrix* genome was submitted to GenBank using the following accession number: CP168152.1, CP168153.1. The strain has been deposited in the “Centre de Ressources Biologiques de l’Institut Pasteur” (CRBIP) under accession number CIP 112583, and in the “Leibniz Institute DSMZ” under accession number DSM 120327.”

The original Article has been corrected.

