# Supplementary material for: Novel insights into insect mediated polystyrene biodegradation through bacterial genome analyses
Source: Sci Rep. 2025 Jan 7;15:1047. doi: 10.1038/s41598-025-85517-x (PMC11707134; doi:10.1038/s41598-025-85517-x)
Supplement: Supplementary file 1 — Supplementary Figures. [file 41598_2025_85517_MOESM1_ESM.pdf]

SUPPLEMENTARY FIGURES

Supplementary Figure 1. Prophage region in the plasmid of *Stenotrophomonas indicatrix* strain DAI2m/c spanning 6942 bp (start: 1, stop: 6943), identified through analysis with PHASTEST. The annotations include 15 phage-related proteins involved in lysis or envelope functions, as well as 5 hypothetical proteins. a) Circular representation of the plasmid. b) Linear representation of the plasmid.

a

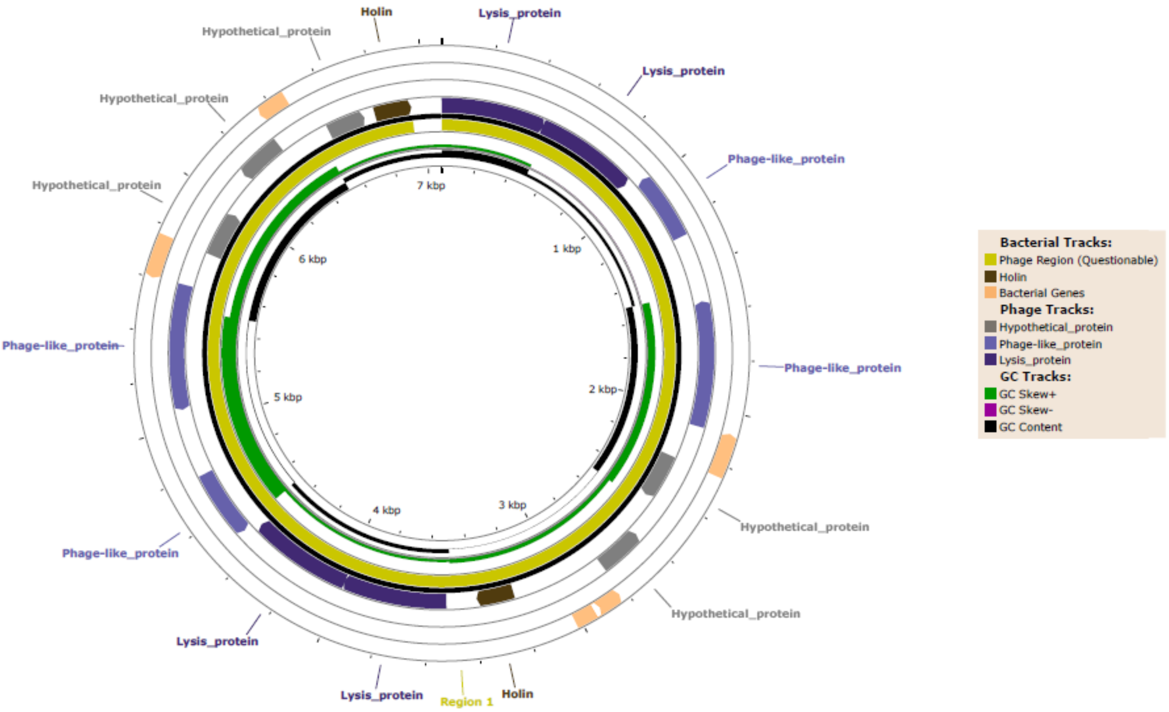

b

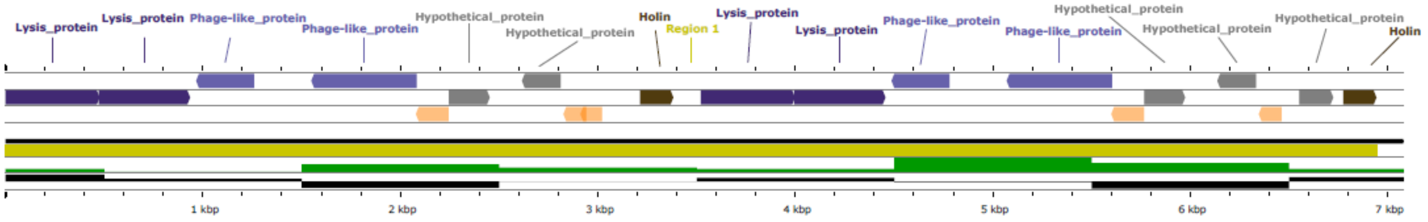

Supplementary Figure 2. Representation of the styrene degradation pathway in *Stenotrophomonas indicatrix* strain DAI2m/c following the original map00634 available in the KEGG database and the Styrene Pathway Map present in BBD. a) The styrene degradation map obtained through automatic functional annotation by assigning orthologous groups via eggNOG; b) The styrene degradation map derived from manual functional annotation using Pfam domains. In both automatic and manual annotations, the presence and absence of styrene map-related enzymes are represented by blue and pink boxes, respectively. In the manual annotation, yellow boxes indicate the enzymes with partial retrieval of Pfam profiles.

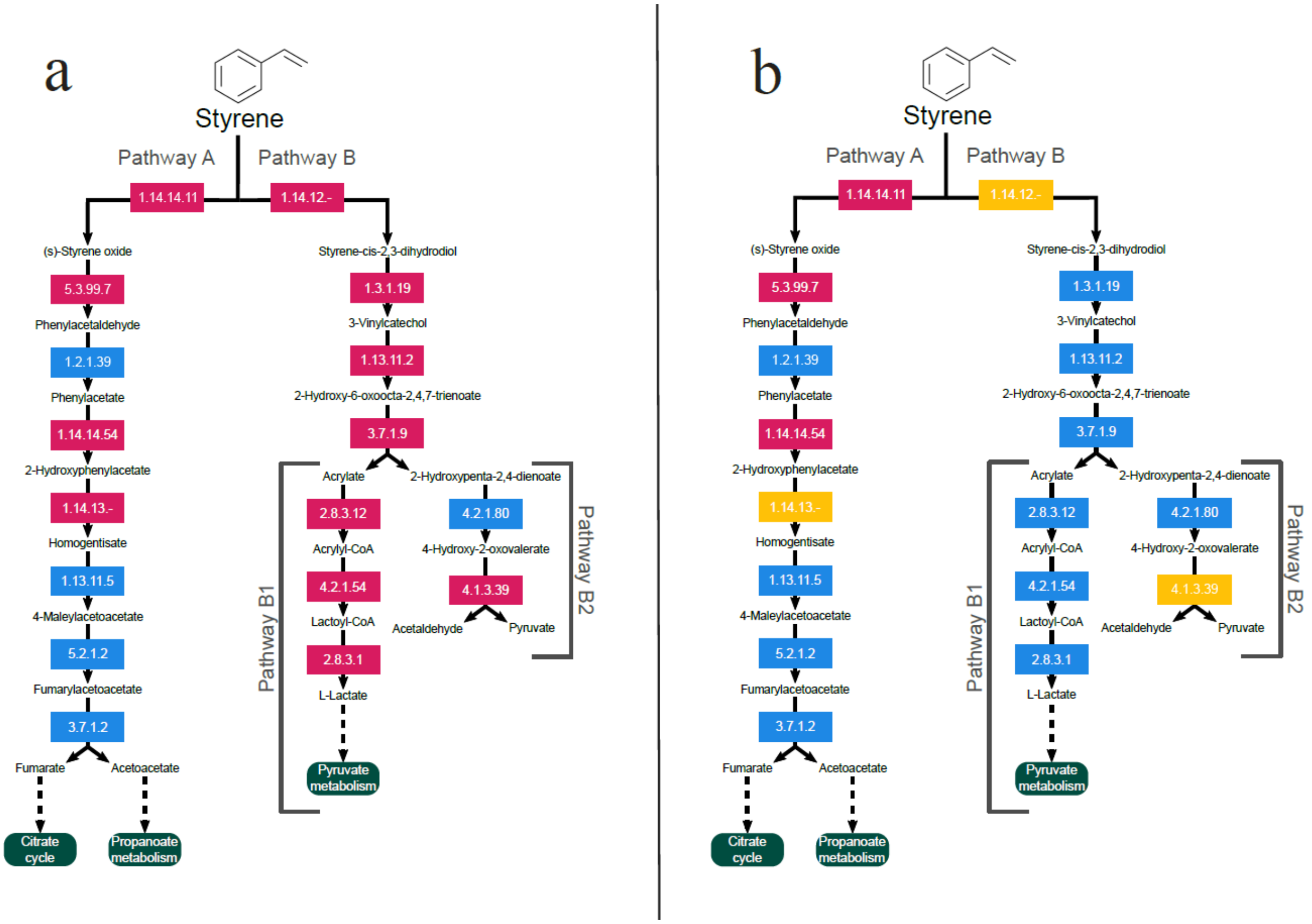

Supplementary Figure 3. Circular representation of the comparative genome analysis between *Stenotrophomonas indicatrix* strain DAI2m/c and *Stenotrophomonas indicatrix* strain DAIF1. The BLAST Ring Image Generator (BRIG) was used to visualize genomic similarities and differences, with *S. indicatrix* DAIF1 serving as the reference genome. The rings represent the genomes of the two strains. The colour gradient indicates the percentage of identity of each gene in the *S. indicatrix* DAIF1, compared to those in *S. indicatrix* strain DAI2m/c. Specifically, the genes of interest involved in the styrene degradation pathway, identified through automatic and manual annotation (excluding those in the yellow boxes, see Fig. S2), are marked. The percentage identities of these enzyme-encoding genes are as follows: 4.2.1.80 (96%), 1.3.1.19 (94%), 2.8.3.1 (97%), 3.7.1.9 (91%), 3.7.1.2 (96%), 5.2.1.2 (93%), 2.8.3.12 (95%), 1.13.11.2 (96%), 4.2.1.54 (98%), 1.2.1.39 (93%), 1.13.11.5 (96%).

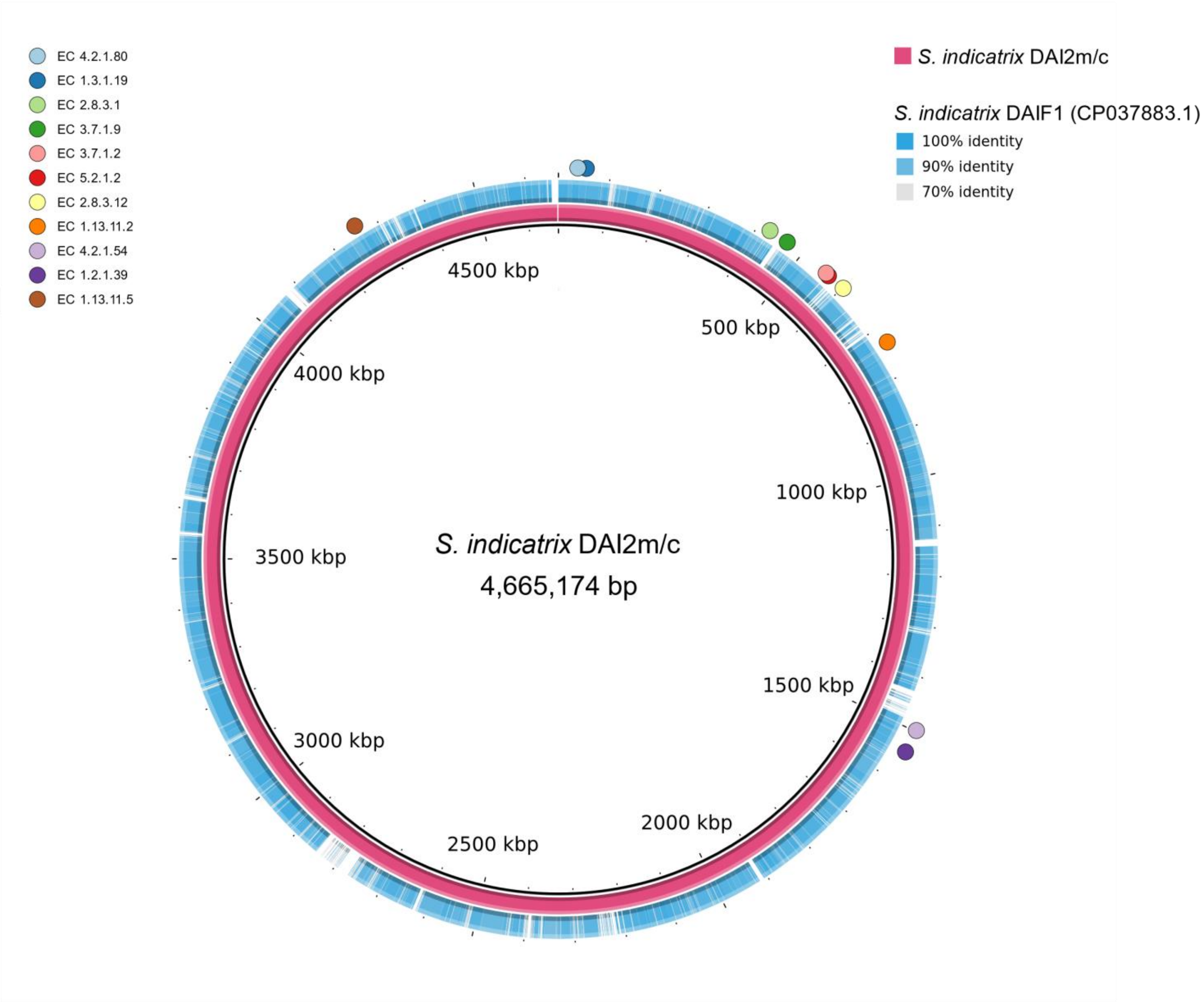

Supplementary Figure 4. Phylogenetic reconstruction of the order Xanthomonadales using BUSCO scOGs, rooted on the Rhodanobacter clade accordingly to Naushad et al. (2015) results. The two families are colour coded. Supported nodes (UFB > 95) are identified with a grey dot. A red arrow indicates the position of S. indicatrix strain DAI2m/c.

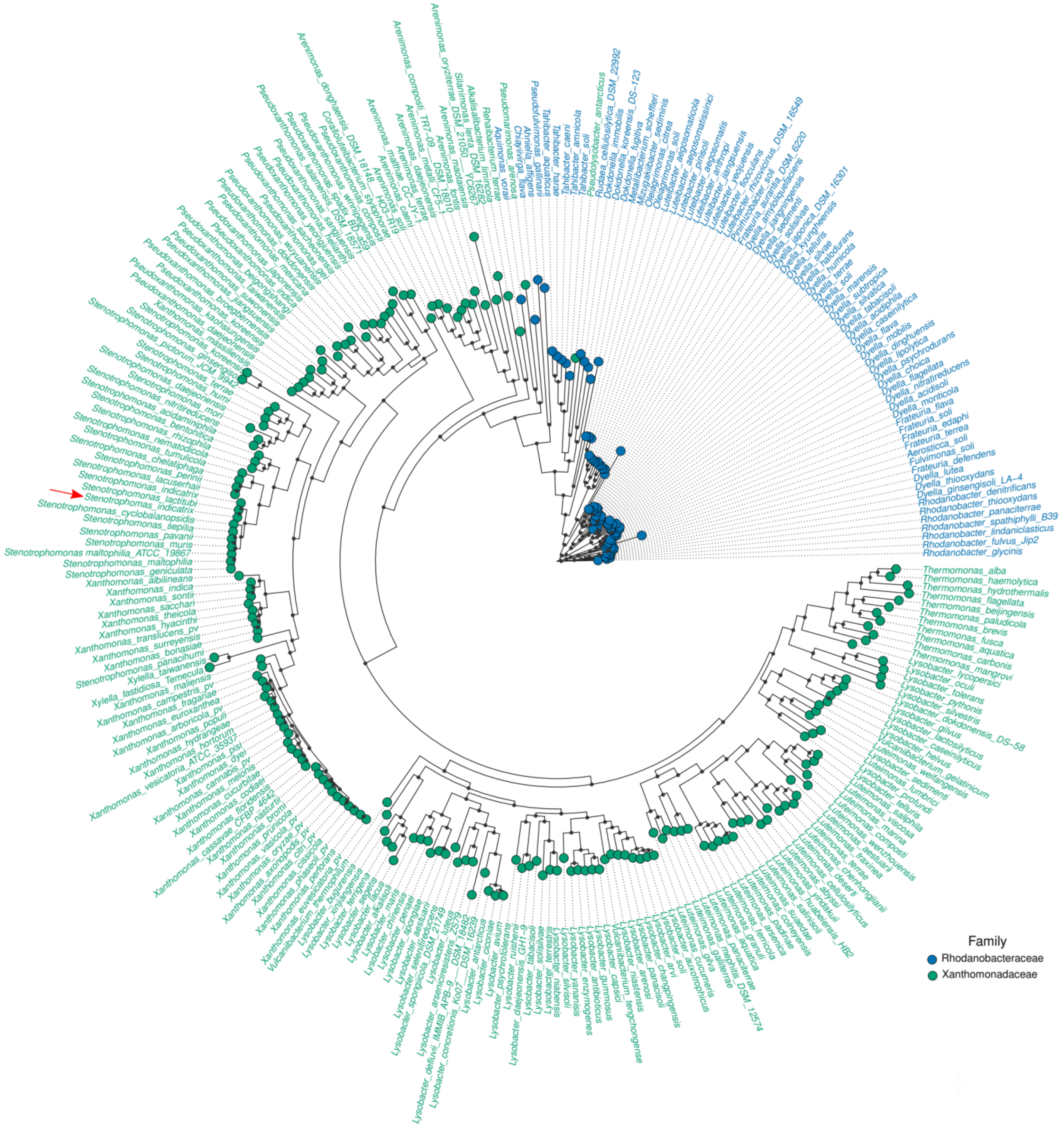

Reference:

Naushad, S., Adeolu, M., Wong, S., Sohail, M., Schellhorn, H. E., & Gupta, R. S. (2015). A phylogenomic and molecular marker based taxonomic framework for the order Xanthomonadales: proposal to transfer the families Algiphilaceae and Solimonadaceae to the order Nevskiales ord. nov. and to create a new family within the order Xanthomonadales, the family Rhodanobacteraceae fam. nov., containing the genus Rhodanobacter and its closest relatives. *Antonie van Leeuwenhoek*, *107*, 467-485.
